# Synthesis, Multinuclear NMR Characterization and Dynamic Property of Organic–Inorganic Hybrid Electrolyte Membrane Based on Alkoxysilane and Poly(oxyalkylene) Diamine

**DOI:** 10.3390/membranes2020253

**Published:** 2012-06-13

**Authors:** Diganta Saikia, Yu-Chi Pan, Hsien-Ming Kao

**Affiliations:** Department of Chemistry, National Central University, Chung-Li 32001, Taiwan; Email: diganta_s@yahoo.com (D.S.); yuchipan421@gmail.com (Y.-C.P.)

**Keywords:** organic–inorganic hybrid electrolyte, ionic conductivity, poly(oxyalkylene) diamine, segmental motion

## Abstract

Organic–inorganic hybrid electrolyte membranes based on poly(propylene glycol)-*block*-poly(ethylene glycol)-*block*-poly(propylene glycol) bis(2-aminopropyl ether) complexed with LiClO_4_ via the co-condensation of tetraethoxysilane (TEOS) and 3-(triethoxysilyl)propyl isocyanate have been prepared and characterized. A variety of techniques such as differential scanning calorimetry (DSC), Fourier transform infrared (FTIR) spectroscopy, alternating current (AC) impedance and solid-state nuclear magnetic resonance (NMR) spectroscopy are performed to elucidate the relationship between the structural and dynamic properties of the hybrid electrolyte and the ion mobility. A VTF (Vogel-Tamman-Fulcher)-like temperature dependence of ionic conductivity is observed for all the compositions studied, implying that the diffusion of charge carriers is assisted by the segmental motions of the polymer chains. A maximum ionic conductivity value of 5.3 × 10^−5^ Scm^−1^ is obtained at 30 °C. Solid-state NMR results provide a microscopic view of the effects of salt concentrations on the dynamic behavior of the polymer chains.

## 1. Introduction

The rapid need for green energy systems due to depletion of fossil fuels increases attention towards lithium ion battery, as it is viewed as one of the most efficient power sources for electric vehicles (EVs) and hybrid electric vehicles (HEVs). However, these batteries have not been applied significantly in EVs or HEVs because of safety issues such as short-circuiting, flaming or thermal runaway, since most of the present available lithium ion batteries employ liquid electrolytes. Therefore, it is highly desirable to have a stable electrolyte for this type of lithium battery without the use of organic solvents.

Solid polymer electrolytes (SPEs) have long been the subject of intense research because of their potential electrochemical applications in solid-state rechargeable lithium batteries, dye-sensitized solar cells, chemical sensors, data storage and electrochromic devices [1,2,3,4,5]. These SPEs are specifically investigated due to their non-flammable, leakage free and flexible characteristics. Flexible structure can accommodate the shape changes during charge-discharge cycle of battery. The discovery of ionic conductivity in polyether based hosts, especially in poly(ethylene oxide) (PEO) and its derivatives, complexed with alkali metal salts by Wright’s group has generated research activities and led to significant advances in characteristics and structure of these polymer-salt complexes [6,7]. However, PEO-based polymer electrolytes show comparatively low ionic conductivity (~10^−7^–10^−8^ Scm^−1^) at ambient temperatures because of the existence of crystalline domains, which interfere with the ionic transport. Different types of polymer based electrolytes, such as co-polymers, blends, materials doped with micron/nano size particles, gel/plasticized polymer electrolytes, *etc*. have been synthesized and succeeded to increase the ionic conductivity of the systems [8,9,10,11,12,13]. It is possible to obtain ionic conductivity in the range of 10^−3^–10^−2^ Scm^−1^ for gel/plasticized polymer electrolytes. The presence of higher amounts of plasticizer in gel polymer electrolytes, however, makes them mechanically poor and leakage prone, which deteriorate the performance of the battery.

Recently, organic–inorganic hybrid electrolytes, so-called ormolytes (organically modified electrolytes) are gaining more attention because of the flexibility in choosing the same host matrix for solid and plasticized polymer electrolytes [14,15,16,17,18,19,20,21,22,23,24]. The organic–inorganic hybrid electrolytes are often prepared via a sol-gel synthetic route through the use of functionalized organosilanes to combine with the polymer backbones. In this way, a relatively stable matrix can be readily tailored to obtain the desired chemical and physical properties. The incorporation of a silicate network into the polymer matrix permits not only a significant reduction or even suppression of crystallinity but also results in the improvement of mechanical and chemical/thermal stability of the materials. Moreover, the in-situ formation of inorganic components within the polymer matrix gives the electrolytes a composite nature which may further help in ionic conduction. Therefore, the hybrid polymer electrolytes with controlled chemical and physical properties are promising candidates to overcome the disadvantages associated with conventional PEO-based electrolytes.

**Scheme 1:**
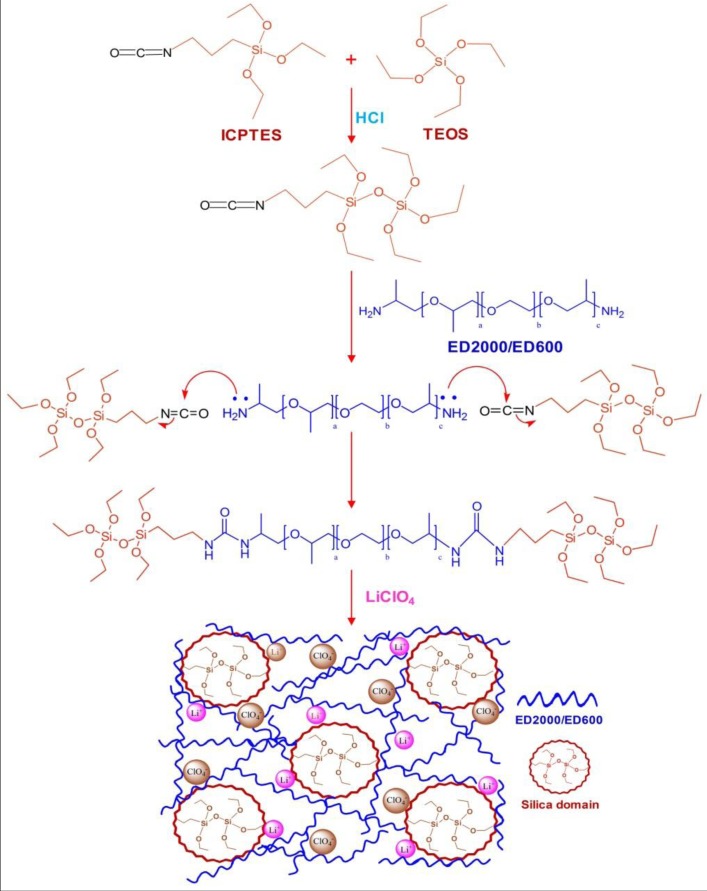
Schematic representation of the synthesis and structure of the present hybrid electrolyte.

With the aim of synthesizing a flexible and robust matrix based hybrid electrolyte, herein 3-isocyanatepropyltriethoxysilane (ICPTES) has been reacted with tetraethoxysilane (TEOS) and then hydrolyzed to bridge the silica network. Condensation and polymerization of this silica network with poly(propylene glycol)-*block*-poly(ethylene glycol)-*block*-poly(propylene glycol) bis(2-aminopropyl ether) (H_2_N-PPG-PEG-PPG-NH_2_) triblock copolymer and doped with LiClO_4_ salt leads to the organic–inorganic hybrid electrolyte. The N=C=O functionality in ICPTES provides a cross-linking center to interact with the amine end groups of the triblock copolymer to generate a semicrystalline diureasil based hybrid. The hybrid electrolytes were designated as TIE(Y)-Z, where T represents the TEOS, I for ICPTES, E stands for the parent triblock co-polymer with Y corresponds to the average molecular weight, either 2000 or 600, and Z (salt composition) indicates the number of ether oxygen atoms (only for polymer) per Li^+^ cation. The synthesis procedures of the organic–inorganic hybrid electrolyte are illustrated in Scheme 1. The microscopic molecular behavior of the hybrid can be analyzed by solid-state NMR spectroscopy. Therefore, the present work highlights the use of multinuclear (^13^C, ^29^Si, ^7^Li) solid-state NMR techniques in order to gain more insights into the ion-polymer interactions, the nature of charge carrier, and the ionic association process in the hybrid electrolyte. Differential scanning calorimetry (DSC), Fourier transform infrared spectroscopy (FTIR) and AC impedance measurement were carried out to study the structural properties, ionic interactions and ionic conductivity of the organic–inorganic hybrid electrolyte, respectively.

## 2. Results and Discussion

### 2.1. Thermal Behavior

**Figure 1:**
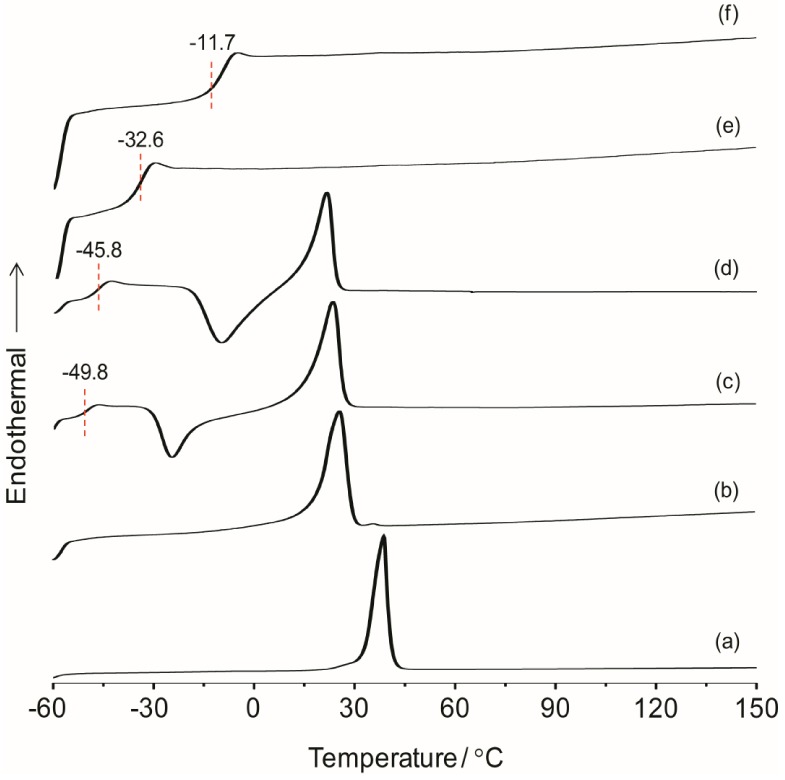
Differential scanning calorimetry (DSC) thermograms of (**a**) pure ED2000 and TIE(2000)-Z hybrid electrolytes with various [O]/[Li] ratios, where Z = (**b**) ∞; (**c**) 32; (**d**) 24; (**e**) 16 and (**f**) 8.

The overall thermal properties of the organic–inorganic hybrid polymer electrolyte systems have been investigated by the differential scanning calorimetry (DSC) technique. The glass transition temperature (*T*_g_), melting point (*T*_m_) and the crystallinity (*χ*) of the material are all important parameters resulting from the microstructure and morphology of the system. These parameters have influence on the overall separator properties of the hybrid electrolyte material when operating in a battery. The thermal behaviors of the hybrid electrolytes TIE(2000)-Z and TIE(600)-Z, with various [O]/[Li] ratios are shown in Figure 1 and Figure S1 (Electronic Supplementary Information, ESI), respectively and the results are summarized in Table 1. As seen in Figure S1, the thermograms of TIE(600)-Z hybrid electrolytes are completely amorphous. The DSC thermograms for TIE(2000)-Z hybrid electrolytes show that the crystallinity of the hybrid decreases with decreasing the [O]/[Li] ratio and become completely amorphous for [O]/[Li] ratios 16 and 8, since no melting transitions are observed. The parent polymer ED2000 has a melting transition at 38.7 °C. After reacting with ICPTES and TEOS, the peak shifts to 25.9 °C. The melting peak further shifts to 23.4 °C and 21.6 °C after doping with LiClO_4_ for the [O]/[Li] ratios of 32 and 24, respectively. The decrease and disappearance of *T*_m_ and the endothermic heat with increasing salt concentrations indicates that the crystalline structure of ED2000 is disrupted and completely suppresses with high level of LiClO_4_ doping. The percentages of the crystallinity of the hybrid electrolytes are estimated using the equation:

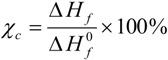
(1)
where 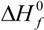 is the enthalpy of fusion of pure ED2000 (111.7 J g^−1^) and *∆H_f_* is the enthalpy of fusion of the hybrid electrolyte and the results are listed in Table 1. As seen in Table 1, the endothermic heat decreases with the decrease in the [O]/[Li] ratios and so as the crystallinity. It suggests that the increased interactions between the ether oxygen atoms of PEG and PPG and the Li^+^ ions with increasing salt concentration inhibit the effective reorganization of the polymer chains and thus help to restrict crystallinity [25]. An exothermic peak in the temperature range of −30 to −10 °C, corresponding to the recrystallization of ED2000 was observed for the TIE(2000)-Z (Z = 32 and 24) samples, while such a peak was not found in the other samples with higher salt contents [22]. The recrystallization phenomenon was also not observed with the TIE(2000)-∞ (no salt) sample. This implies that the recrystallization process is related with lower salt concentration and it is suppressed by higher salt contents for the other hybrid electrolyte samples (TIE(2000)-16 and 8).

**Table 1:** Glass-transition temperature (*T*_g_), melting temperature (*T*_m_), endothermic heat (Δ*H_f_*), crystallinity (*χ*_c_), Fourier transform infrared (FTIR) deconvolution result, Arrhenius activation energy (*E*_a_) and Vogel-Tamman-Fulcher (VTF) parameters of TIE(Y)-Z hybrid electrolytes with various [O]/[Li] ratios.

Samples	TIE (2000)-Z								TIE (600)-Z	
	*T*_g_ (°C)	*T*_m_ (°C)	Δ *H*_f _(J g^−1^)	*χ*_c_ (%)	Free ClO_4_^−^, % (~625 cm^−1^)	*E*_a_ (eV)	*A* (Scm^−1^ K ^0.5^)	*B* (eV)	*T*_g_ (°C)	Free ClO_4_^−^, %
Z										
Pure ED2000	-	38.7	111.7	100	-	-	-	-	-	-
∞	-	25.9	56.6	50.6	-	-	-	-	−31.7	-
32	−49.8	23.5	39.9	35.5	100	0.32	36.7	0.13	−25.5	85.2
24	−45.8	21.6	29.3	26.3	88.2	0.3	60.2	0.12	−20.0	81.3
16	−32.6	-	-	-	85.7	0.35	141.6	0.12	−11.5	79.3
8	−11.7	-	-	-	75	0.42	60.5	0.1	7.8	73.3

The mobility of the Li ions within the polymer matrix is one of the factors which govern the ionic conductivity in SPEs. It depends on the segmental motion of the polymer chains in the amorphous phase, which is characterized by *T*_g_. *T*_g_ is one of the most important parameters of the amorphous phase for the flexibility of the polymer at room temperature. As seen in Figure 1 and Figure S1, the *T*_g_ values are progressively shifted to higher values with increasing salt concentrations for the TIE(2000)-Z and TIE(600)-Z hybrid electrolyte systems. The increase in the *T*_g_ value is attributed to the interactions developed between the polymer segment and the salt due to the formation of transient cross-links between the salt and the polyether phase [26]. As the salt concentration increases in the hybrid electrolytes, the formation of associated ionic species (e.g., contact ion pairs and/or ion aggregates) also increases and these aggregates may be less mobile than the dissociated ions. The presence of such polymer-salt interactions restricts segmental mobility of the host hybrid matrix and progressively increases the *T*_g_ values.

The polymer chain length also influences the *T*_g_ of the hybrid matrix. As seen in Table 1, the increase of the PEG fraction of the polymer chains in TIE(2000)-Z decreases the *T*_g_ values more for all the lithium concentrations in comparison to TIE(600)-Z hybrid electrolytes. This suggests that the PEG fraction of the polymers has an effect on the dynamics of the polymer and influence the *T*_g_. Since the polymer chains are bonded to the silicate network by covalent bonds, it is reasonable that the entire chain is closer to the silica node and the segmental motions are thus more hindered for the case of shorter PEG chains. For longer PEG chains, however, only a small fraction of the chains is located near the silica interface and the majority of chain segments have high mobility, which makes TIE(2000)-Z hybrids more flexible and lower *T*_g_ values than TIE(600)-Z hybrids. 

### 2.2. Polymer-Ion Interactions

**Figure 2:**
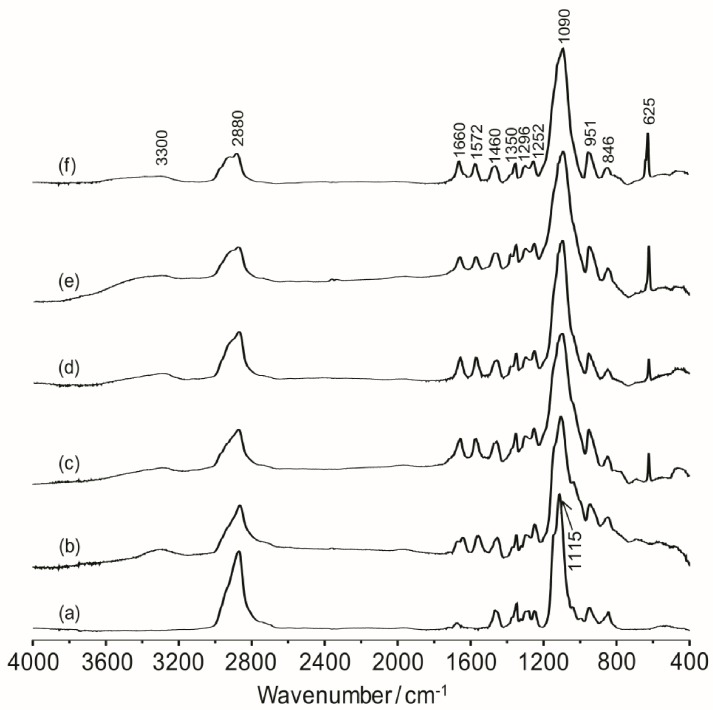
FTIR spectra of (**a**) pure ED2000 and TIE(2000)-Z hybrid electrolytes with Z = (**b**) ∞; (**c**) 32; (**d**) 24; (**e**) 16 and (**f**) 8.

Infrared spectroscopy is a convenient method to provide the information about the structure of organic–inorganic system and the interaction of the polyether with the lithium salt. Figure 2 shows the FTIR spectra of the TIE(2000)-Z hybrid electrolytes with various [O]/[Li] ratios. The band observed around 3300 cm^−1^ is assigned to the hydrogen-bonded N–H stretching mode [27]. The band around 2880 cm^−1^ is due to –CH_2_ stretching of polyether groups [28]. The band at 1660 cm^−1^ is assigned to the amide I modes with hydrogen bonding. Generally, the amide I band is related to C=O stretching, the C–N stretching and C–C–N deformation vibrations [29]. The amide II band is associated with the vibration of N–H in-plane bending, the C–N stretching, and the C–C stretching modes [29,30]. The band observed at 1572 cm^−1^ is attributed to the amide II mode. The presence of these amide bands confirms a successful synthesis of the hybrid. The peaks at 1460, 1350, 1296, 1252 and 846 cm^−1^ are assigned to –CH_2_ bending, wagging, twisting and rocking vibrations of the polymer, respectively [31,32]. One major peak associated with C–O–C asymmetric stretching vibration is observed at 1115 cm^−1^ for pure ED2000. After formation of the polymer-silica hybrid (*i.e.*, TIE(2000)-∞), the peak becomes broader since the characteristic absorption bands of the hydrolysis product of TEOS and ICPTES are also expected to appear in the same region. With the addition of the lithium salts, the peak at 1115 cm^−1^ is shifted to 1090 cm^−1^, suggesting that the ether groups of ED2000 have some interactions with the added lithium cations. The typical absorption band for noncondensed Si–OH is observed around 951 cm^−1^ [33].

**Figure 3:**
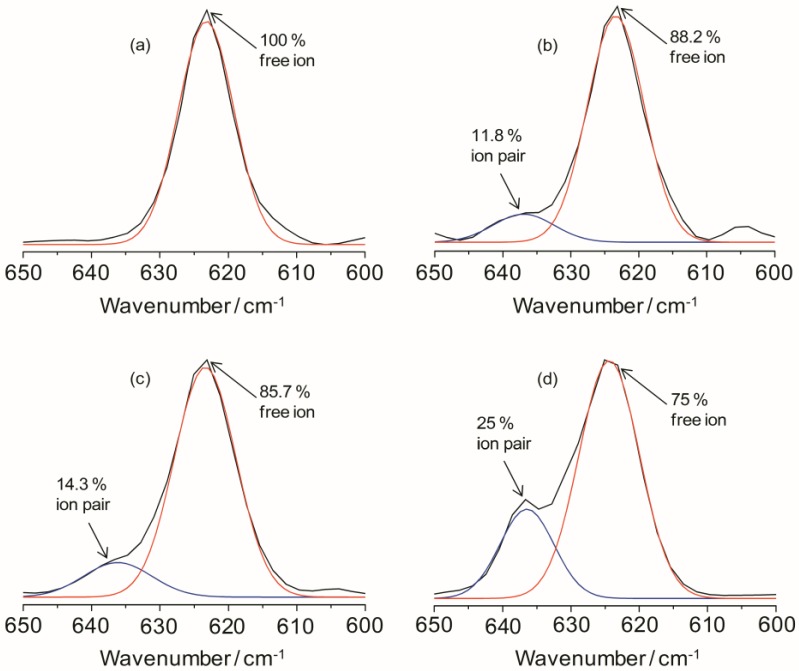
IR deconvolution results (in the range of 600 to 650 cm^−1^) of TIE(2000)-Zhybrid electrolytes with various [O]/[Li] ratios, where Z = (**a**) 32; (**b**) 24; (**c**) 16 and (**d**) 8.

The ionic association of the hybrid electrolyte system can be analyzed by estimating the vibrational modes of the ClO_4_^−^ anion. The characteristic *ν*(ClO_4_^−^) mode of LiClO_4_ is particularly sensitive in changing the ion-ion interactions in the electrolyte systems. The peak around 610 to 650 cm^−1^ in the IR spectra for the TIE(2000)-Z samples can be deconvoluted into two bands centered at 625 and 635 cm^−1^ due to the vibration modes of ClO_4_^−^ ions. The band around 625 cm^−1^ has been assigned to the vibration of the “free” ClO_4_^−^ anion, which does not interact directly with the lithium cations, and the band around 635 cm^−1^ to the vibration of the Li^+^ClO_4_^−^ contact-ion pairs [34,35]. The behavior of ionic association is investigated by fitting the *ν*(ClO_4_^−^) envelop with Gaussian-Lorentzian functions and the results are displayed in Figure 3 for TIE(2000)-Z hybrid electrolytes. The fraction of “free” anions and contact-ion pairs has been calculated as the ratio of the area under the peaks attributed respectively to “free” anions and contact-ion pairs to the total area for the *ν*(ClO_4_^−^) vibrations. As can be seen in Figure 3, the peak characteristic for “free” anions is much larger than that representing contact-ion pairs. The fractions of “free” anions as a function of salt concentration for the hybrid electrolytes are given in Table 1. As seen in Table 1, the fraction of free anions decreases with increasing salt concentrations. About 100% of ClO_4_^−^ exists as spectroscopically “free” species for TIE(2000)-32, while about 75% of free ClO_4_^−^ is observed for the electrolyte TIE(2000)-8, with higher salt concentration (Table 1). This is as expected since more and more free ions become bound with the opposite ions to form contact ions upon addition of more salts leading to the decrease in free ions. The degree of ion dissociation slightly increased when the molecular weight of the polymer host changed from 600 to 2000 g mol^−1^ (Table 1). As the [O]/[Li] ratios are based on the PEO and PPO unit in the oligomers (ED2000, PEO = 40.5 and PPO = 3.5; ED600, PEO = 8.5 and PPO = 3.5) and amount of PPO is same in both the oligomers, the slight increase in degree of ion dissociation at a given [O]/[Li] ratio may be due to the contribution from the PEO segment. Since PPO is expected to have a poorer ability to dissolve salts as compared to PEO, these hybrid systems based on the polymer ED2000 have a high degree of ionic dissolution, suggesting that the effect of the silica network resulting from the sol-gel condensation of silica precursors is important as in the case of composite polymer electrolytes.

### 2.3. Structure Elucidation by ^13^C CPMAS NMR

**Figure 4:**
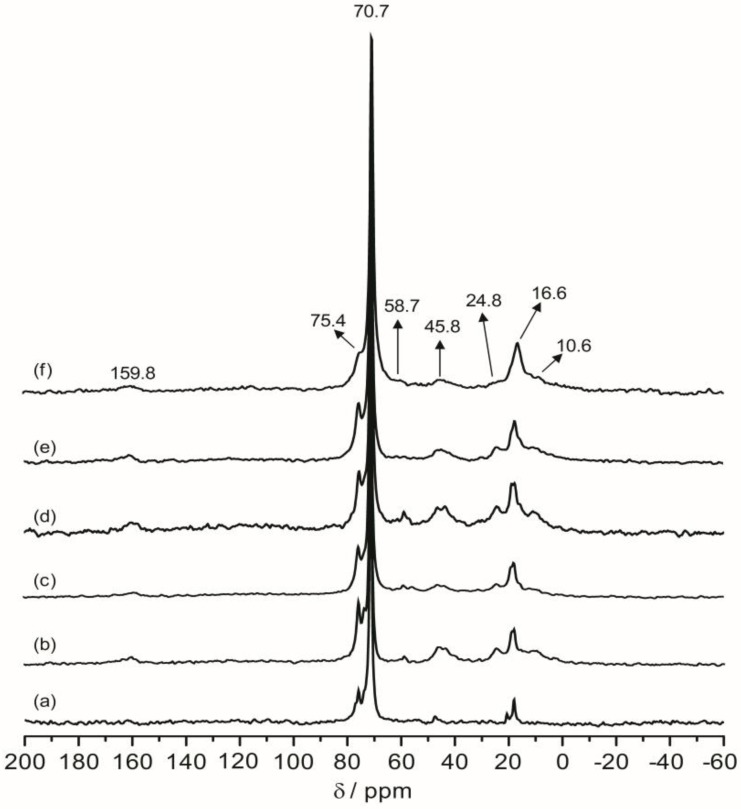
^13^C CPMAS NMR spectra of (**a**) pure ED2000 and TIE(2000)-Z hybrid electrolytes with Z = (**b**) ∞, (**c**) 32, (**d**) 24, (**e**) 16 and (**f**) 8.

Solid-state ^13^C CPMAS NMR was performed to obtain the backbone structure of the hybrid electrolytes. Figure 4 shows the ^13^C CPMAS NMR spectra of the pure ED2000 and TIE(2000)-Zhybrid electrolytes with various [O]/[Li] ratios. The most predominant peak at 70.7 ppm is assigned to methylene carbons adjacent to the ether oxygen of the polymer chain. The methyl carbon from the propylene oxide units appear at 16.6 ppm, while the peak at 45.8 ppm can be assigned to the nitrogen substituted carbons at the terminal of the diamines. Two peaks at 24.8 and 10.6 ppm are assigned to the methylene carbons in the α and β positions to the silicon atom of ICPTES, respectively, while a small peak at 58.7 ppm is due to the non-hydrolyzed ethoxy groups in organosilanes. Besides the major peak at 70.7 ppm, there is a smaller peak at 75.4 ppm due to the ether carbons in the PPG segments, which is also clearly resolved for the parent ED2000. This peak become a broader shoulder and is no longer resolved when the salt doping level reaches the maximum. The high level of salt doping causes a more broad distribution of the environments of the polymer chains, which results in a broader low-field shoulder near the peak at 70.7 ppm. The urea functional groups of this hybrid matrix, which are considerably less abundant, give rise to a very weak signal at 159.8 ppm of the Li^+^-doped hybrid electrolytes. This confirms the formation of urea linkages between the polymer diamine and ICPTES. The CPMAS NMR observations are consistent with the hybrid structure as illustrated in Scheme 1.

### 2.4. Silicate Architecture

**Figure 5:**
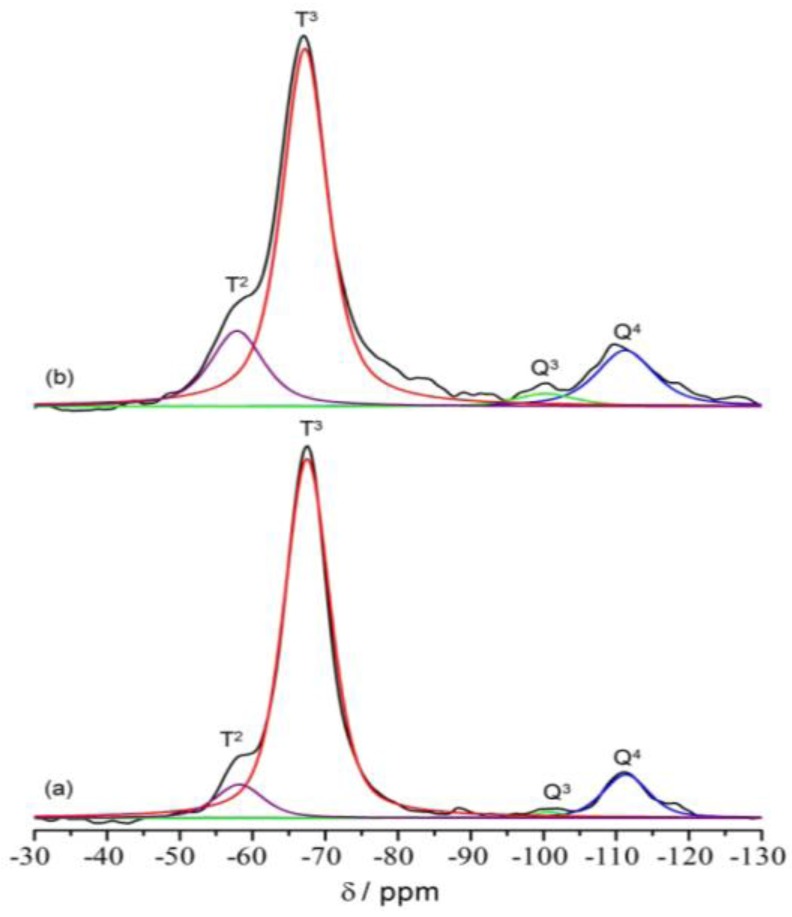
^29^Si MAS NMR spectra of (**a**) TIE(600)-32 and (**b**) TIE(600)-8 hybrid electrolytes. Different color lines represent the components used for spectral deconvolution.

^29^Si MAS NMR is a direct method to characterize the silicon condensation and to indicate the silica network architecture inside these hybrid materials. As shown in Figure 5, four major signals at −67.2 and −58.5 ppm, corresponding to *T*^3^ (R*Si*(OSi)_3_, R = alkyl group) and *T*^2^ (R*Si*(OSi)_2_(OH)) sites, and at −110.1 and −100.3 ppm, corresponding to *Q*^4^ (*Si*(OSi)_4_) and *Q*^3^ (*Si*(OSi)_3_(OH)) sites are observed for the two selected TIE(600)-32 and TIE(600)-8 samples. The spectral features for all samples are basically the same, but the populations of various silicon environments (*T*^2^*vs. T*^3^) show some changes as a function of the [O]/[Li] ratio. The *T*^2^/*T*^3^ ratio changes from 9.7% for TIE(600)-32 to 24.6% for TIE(600)-8, indicating that the condensation of the silicate network is more complete with lower salt concentrations. The presence of both *T* and *Q* groups in the samples indicates that the silicate network is formed in the hybrid as shown in Scheme 1.

### 2.5. ^13^C CPMAS NMR with Variable Contact Time

**Figure 6:**
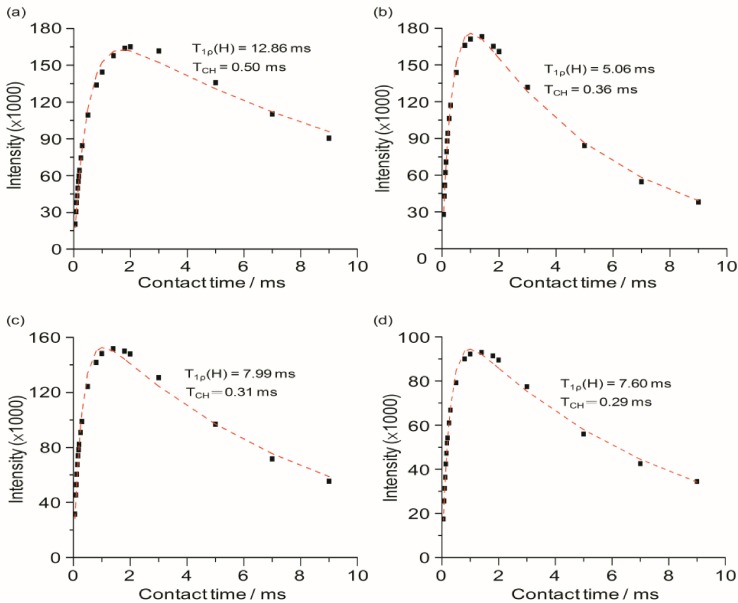
^13^C CPMAS NMR spectra (70.7 ppm) of TIE(600)-Z hybrid electrolytes with Z = (**a**) 32; (**b**) 24; (**c**) 16 and (**d**) 8, as a function of contact time.

Solid-state ^13^C CPMAS NMR experiments performed with various contact times are useful to gain more insights into the influence of Li^+^ ions on the local dynamics of the polymer chains from the microscopic view. The ^1^H → ^13^C CP signal intensity for the ether carbon at 70.7 ppm as a function of contact time and salt doping levels is chosen for the analysis. The ^13^C CPMAS NMR with variable contact time for TIE(600)-Z system is depicted in Figure 6. The *T*_CH_ and *T*_1_*ρ*(H) measurements are obtained by fitting the CP signal intensities with the following equation [36]:

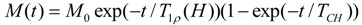
(2)
where *M*(*t*) is the peak intensity as a function of contact time *t*, *M*_0_ is the normalization constant, *T*_1_*ρ*(H) is the proton spin-lattice relaxation in the rotating frame and *T*_CH_ is the cross-polarization time constant. Since CP is a measure of the efficiency of magnetization transfer by the dipolar coupling from ^1^H to ^13^C spins, it is most efficient for the static ^1^H–^13^C dipolar interactions. As a result, the less mobile carbon groups exhibit the faster cross-polarization rate or the shorter *T*_CH_. The slower growth in spin magnetization for the peak at 70.7 ppm for TIE(600)-32 (*T*_CH_ = 0.50 ms), in comparison to that of TIE(600)-8 (*T*_CH_ = 0.29 ms), is consistent with its higher mobility of polymer chains, as also indicated by its low *T*_g_. This reveals that the rapid motion of the TIE(600)-32 makes the CP signal transfer from the proton spins less efficient than for the TIE(600)-8. The significant decrease in *T*_CH_ for the peak at 70.7 ppm with increasing salt concentration suggests a possible coordination of the Li^+^ ions with the ether oxygen atoms of the polymer chain, thereby restricting the segmental motion and leading to stronger ^1^H–^13^C dipolar couplings. The *T*_1_*_ρ_*(H) value for the peak at 70.7 ppm decreases as the salt concentration is increased, reflecting that proton–proton spin diffusion becomes more efficient due to the increased proton–proton dipolar interaction. The coordination between the Li^+^ ions and the polyether chains effectively reduces the chain motion, resulting in the chain rigidity, and therefore increases proton–proton dipolar interactions. 

### 2.6. 2D ^1^H–^13^C WISE NMR

**Figure 7:**
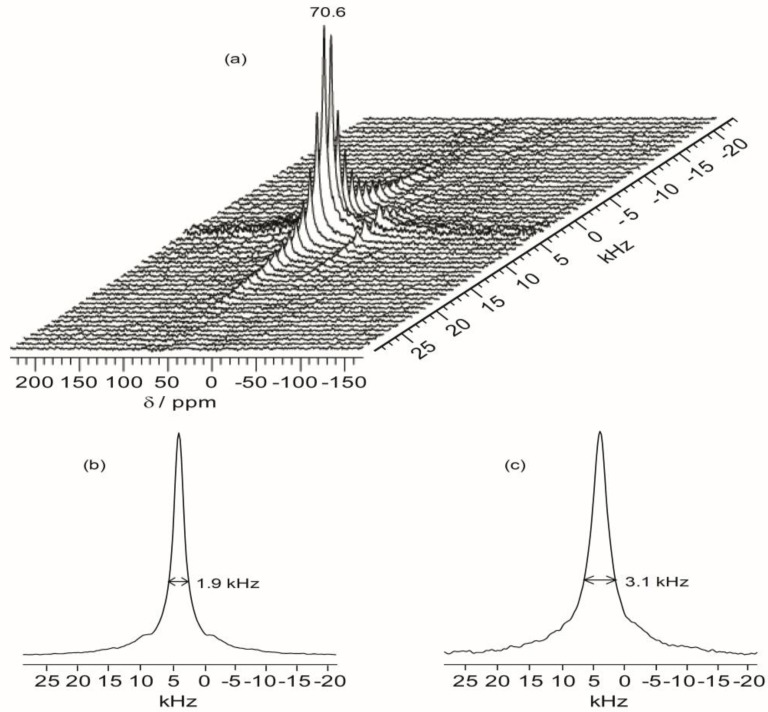
Representative 2D ^1^H–^13^C wide-line separation (WISE) NMR spectrum (**a**) and the projections of the ^1^H dimension of 2D ^1^H–^13^C WISE spectra associated with the 70.7 ppm peak in the ^13^C dimension for TIE(600)-Z hybrids, where Z = (**b**) 32 and (**c**) 8.

Two-dimensional wide-line separation (WISE) NMR spectroscopy is a simple and elegant method to measure the local mobility of polymers by correlating the proton lineshape with the carbon chemical shift [37]. With this technique, the spectroscopic information about the dynamic behavior within the present hybrid can be qualitatively assessed by examining the proton line widths associated with the ether carbons in the polymer host. Figure 7 shows the projections of the ^1^H dimension of the WISE spectra associated with the 70.7 ppm peak for TIE(600)-32 and TIE(600)-8. The linewidth of the ^1^H line reflects the nature of the dipolar interaction between the protons and thus can be used to monitor the mobility of the polymer chains. For the selected carbon signal at 70.7 ppm, the TIE(600)-8 sample exhibits a larger linewidth (3.1 kHz) in the ^1^H dimension than the TIE(600)-32 sample (1.9 kHz). This indicates some microscopic dynamic changes of the polymer chains as the salt content is increased. 

Thus, the 2D WISE NMR spectra confirm that the mobility of the polymer chains decreases as the salt concentration is increased due to complexation of Li^+^ with the ether oxygen atoms. In comparison to most crystalline or semicrystalline polymers, which always exhibits proton linewidth larger than 50 kHz, the much narrower proton linewidth from the 2D WISE NMR reveals considerable chain mobility for the present hybrid electrolytes. The WISE NMR results provide a microscopic view into the mobility change of the polymer chains as functions of salt concentrations, which are also corroborated by the *T*_g_ trends as revealed by DSC.

### 2.7. Ionic Conductivity

**Figure 8:**
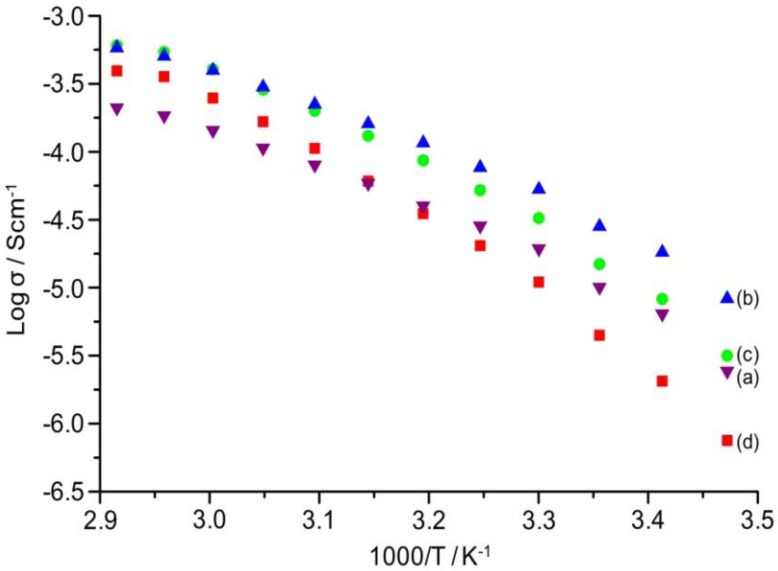
Temperature dependence of ionic conductivity of TIE(2000)-Z hybrid electrolytes with Z = (**a**) 32; (**b**) 24; (**c**) 16 and (**d**) 8.

The temperature dependence of ionic conductivity of the TIE(2000)-Z and TIE(600)-Z organic–inorganic hybrid electrolytes with various [O]/[Li] ratios are shown in Figure 8 and Figure S2 (ESI), respectively. The variation of conductivity with temperatures suggests a Vogel-Tamman-Fulcher (VTF) like behavior, indicating that the ion transport in the present hybrid electrolytes is mainly dependent on the polymer segmental motion. It is observed that ionic conductivity of the hybrid electrolytes increases with increase in salt concentration and reached the maximum at [O]/[Li] = 24. The hybrid electrolyte exhibits the highest ionic conductivity values of 5.3 × 10^−5^ Scm^−1^ and 4.0 × 10^−4^ Scm^−1^ at 30 °C and 60 °C, respectively, for TIE(2000)-24 sample. Further increase in the salt content results in a decrease in the ionic conductivity for the hybrid electrolyte with [O]/[Li] = 16 and 8. The conductivity trend can be described from FTIR data on free ClO_4_^−^ ion. As seen in Table 1, the lithium ions in the TIE(2000)-32 sample are almost 100% free in comparison to 88% in TIE(2000)-24. The total number of lithium ions are less in [O]/[Li] = 32 than [O]/[Li] = 24, which are not enough to push the ionic conductivity value above the conductivity value of [O]/[Li] = 24. For [O]/[Li] = 16 and 8, by contrast, the number of charge carriers is more, but the amount of contact ion pairs/aggregates is also enhanced (14% and 25%, respectively). These aggregates naturally contribute less effectively to charge transport and eventually hinder the mobility of the charge carriers throughout the polymer matrix. Therefore, the optimum ionic conductivity value is obtained for the TIE(2000)-24 sample. The activation energy (*E*_a_) is calculated from the conductivity plot and the values are given in Table 1. As seen in Table 1, the activation energy increases with the increase in salt concentration. The increase in the ion-ion interaction (e.g., formation of ion pairs/multiple ions) with the increase in salt concentration reduces the ionic mobility and thus enhances the activation energy. This result also complies with the FTIR deconvolution results, which show more ion pairs at higher salt concentration. 

As the conductivity plot suggests a curve profile, necessitating interpretation of the results by the VTF equation:

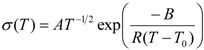
(3)
where *A* is the pre-exponential factor related to the number of charge carriers in the polymer electrolyte, *B* is the pseudo-activation energy of the ion transport related to the configurational entropy of the polymer chains, *R* is the perfect gas constant, 8.314 JK^−1^mol^−1^ and *T*_0_ is the ideal transition temperature at which the configurational entropy becomes zero, *i.e.*, the disappearance of the free volume is complete. *T*_0_ is fixed at *T*_g_ − 50 K for the present electrolytes resulting in a good fit to the data. The VTF equation predicts that a plot of ln(*σT*^0.5^) *vs*. 1/(*T* − *T*_0_) should be linear, which is nearly observed in Figure S3 (ESI). The values of the VTF parameters (*A* and *B*) for the hybrid electrolytes are listed in Table 1. The *A* parameter increases with the increase in salt concentration and exhibits a maximum value for the [O]/[Li] = 16. A further increase in salt concentration decreases the value of *A*. Moreover, the calculated values of the pseudo-activation energy, *B*, are in the range of 0.1–0.13 eV, indicating sufficient mobility of the ions.

In the case of TIE(600)-Z hybrid electrolyte system, the maximum room temperature ionic conductivity value achieved is 4.9 × 10^−6^ Scm^−1^ with [O]/[Li] = 8. It is observed that the ionic conductivity increases with the increase in the salt concentrations. Although the number of free ions are more in the case of [O]/[Li] = 32 (85%), the sample with [O]/[Li] = 8 also possesses more than 73% free ions, which may be sufficient to get this value of conductivity. In addition, the number of charge carriers are more in [O]/[Li] = 8 in comparison to 32, which may assist in the enhancement of ionic conductivity. The conductivity of the TIE(2000)-24 sample is almost one order higher than the conductivity of the TIE(600)-8 sample. This may be attributed to the longer chains of alkylene oxides of ED2000, compared to ED600, and hence the lower *T*_g_ of TIE(2000)-Z based samples. In addition, the higher percentage of ethylene oxide to propylene oxide ratio in ED2000 than ED600 (about 40.5 and 8.5 PEG units contain in ED2000 and ED600, respectively) is also helpful for the enhancement of conductivity [18]. These conductivity results are comparable or higher than the organic–inorganic hybrid electrolytes previously reported [14,15,16,17].

The behavior of conductivity enhancement with temperature can be understood in terms of the free-volume model [38]. As the temperature increases, the vibrational energy of the polymer segments has acquired sufficient energy to push against the hydrostatic pressure imposed by its neighboring atoms and thus polymer can expand easily to produce free volume. Therefore, with the increase in temperature, the free volume increases. The resulting conductivity, represented by the overall mobility of ions and the polymer, is determined by the free volume around the polymer chains. Therefore, as temperature increases, ions, solvated molecules, or polymer segments can move into the free volume [39]. This leads to an increase in ion mobility and segmental mobility that will assist ion transport and virtually compensate for the retarding effect of the ion clouds.

Several factors, such as mobility of polymer chains, cation and anion types, and salt concentration, are important to determine the ionic conductivity in polymer electrolytes. The ionic conductivity of a polymer electrolyte depends on the actual concentration of the conducting species and their mobility and may be given as:

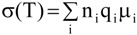
(4)
where n_i_ is the number of charge carriers, q_i_ is the charge on each charge carrier, and μ_i_ is the mobility of charge carriers. According to the equation (4), the ionic conductivity depends on the amount of charge carriers in the system and the mobility of the various species. Mobility of the charge carriers depends on the *T*_g_ of the polymer and it is observed that *T*_g_ increases linearly with increasing salt concentration. The increase in the *T*_g_ can be ascribed to the inter- and intramolecular coordination of ether dipoles with the charge carriers, *i.e.*, dissociated ions, which may act as transient cross-linking points in the polymer electrolytes. The increase in the *T*_g_ decreases segmental motion of the matrix polymer, which directly reduces the ionic mobility.

It has been reported that the number of ions available for conduction is not the only cause for the increase in conductivity exhibited in amorphous and semicrystalline polymers. Ionic conductivity may depend on the influence of the filler on the mobility of both the polymer and the salt [40]. As the present organic–inorganic hybrid electrolyte produces *in*-*situ* silica domains in the matrix, it can be considered as a two-phase system consisting of an ionically conducting polymer matrix with dispersed silica network. The space charge interphase layer between the polymer electrolyte and silica particles makes conductive paths for ions to move easily [41]. At low salt concentration levels, the extent of contact ion pairs is low and the conductivity is dominated by the mobility of charge carriers, which is promoted by the existence of a space-charge layer at the polymer/silica interface. Therefore, the maximum ionic conductivity found for TIE(2000)-24 electrolyte sample is assumed to be caused by the combined effects of segmental movement, charge carrier concentration, *T*_g_ and existence of polymer/silica interface layer in the hybrid.

### 2.8. ^7^Li Linewidth Measurements

The mobility and transport of the charge carriers in a dynamic environment created by the polymer motion in the amorphous phase is a key factor for the ionic conductivity. The static ^7^Li line width measurements as a function of temperature are useful in determining the status of the motional narrowing process of the lithium cations. The NMR spectrum of the ^7^Li spin (*I* = 3/2) in a crystalline sample is expected to consist of a narrow component due to the 1/2 ↔ −1/2 central transition and a Pake doublet due to the 3/2 ↔ 1/2 and –1/2 ↔ −3/2 satellite transitions [36]. At lower temperatures the lineshape consists of a relatively narrow line superimposed on a broader line typically observed in other disordered systems [42]. The broad or quadrupolar component is associated with the satellite transitions (±3/2 ↔ ±1/2), while the narrow component represents the central transition (1/2 ↔ −1/2). When the temperature is increased, both the narrow and broad components are narrowed around the glass transition temperature. Because the present hybrid electrolyte is a heterogeneous system, it is expected that there is a wide distribution of possible electric field gradients (EFG), which results in a Gaussian-broadened satellite line. Since, on the first order, the satellite splitting is dependent on the electric field gradients, the distribution of the quadrupolar coupling constants produced by disorder may smear out the satellites. Chung *et al.* have shown that, at the low temperatures, the width of the central line is independent of the Larmor frequency and concluded that the dipolar interaction is the dominant factor in the 1/2 ↔ −1/2 central transition [43].

The line associated with the 1/2 ↔ −1/2 transition is not broadened (up to the first order) by the quadrupole coupling [44,45], but other broadening sources, such as dipole–dipole couplings to neighboring nuclei, can contribute to its linewidth. Besides the homonuclear dipole–dipole broadening of the central line, there are heteronuclear contributions from protonic species (PEG and PPG) and coupling with the chlorine and oxygen from ClO_4_^−^ species. Given the small gyromagnetic factors and/or low natural abundance of ^13^C, ^35^Cl and ^17^O stable isotopes, one can conclude that the ^7^Li–^7^Li and ^7^Li–^1^H dipolar interactions are two major sources responsible for the observed lithium linewidth.

**Figure 9:**
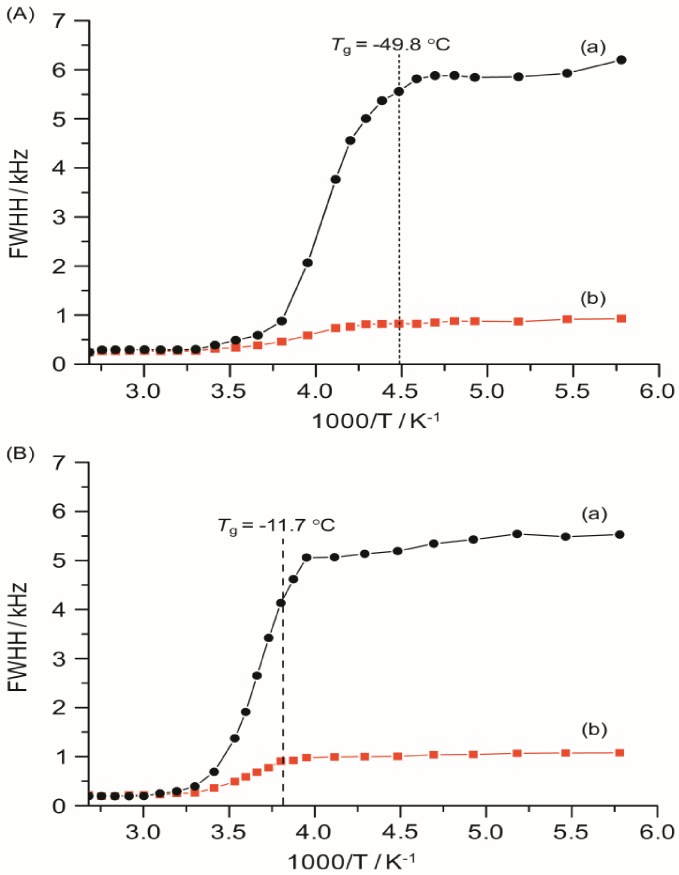
Temperature dependence of ^7^Li static line widths of TIE(2000)-Z hybrid electrolytes with various [O]/[Li] ratios, where Z = (**A**) 32 and (**B**) 8, measured (a) without and (b) with proton decoupling. The dashed lines represent the *T*_g_ values obtained from DSC.

The ^7^Li linewidths, measured with and without ^1^H decoupling, are shown in Figure 9 as a function of temperature from −100 to 100 °C for TIE(2000)-32 and TIE(2000)-8 samples. The static ^7^Li NMR linewidth evolution as a function of temperature can be described by a curve composed of two plateaus separated by a temperature range where a rapid change in the linewidths occurs. At the low-temperature region of −100 to −50 °C, below the *T*_g_ of the systems, the line widths are very broad (fullwidth at half-height (FWHH) ≈ 5–6 kHz) and are not very sensitive to temperature changes. This suggests that the lithium ions are not mobile at low temperatures and thus are not conductive. The broad linewidth observed is the result of increased quadrupolar and/or internuclear dipole-dipole interactions. Upon increasing the sample temperature, the line widths are motionally narrowed, with the onset of narrowing correlating with the *T*_g_. Motional narrowing begins when the rate of the fluctuations (1/τ_c_) of either the local dipolar fields or the electric field gradients (EFG) is comparable to their respective rigid lattice linewidths (Δ_RL_) or when 1/τ_c_ ~ Δ_RL_, where 1/τ_c_ is the motional correlation time [36]. The onset temperature of narrowing for both TIE(2000)-32 and TIE(2000)-8 samples corroborated well with their glass transition temperature, suggesting that the mobility of the lithium cations are coupled with the polymer dynamics in a cooperative way.

From the TIE(2000)-Z spectra it is possible to remove the heteronuclear interaction from the ^7^Li natural linewidth. The ^7^Li natural linewidths for TIE(2000)-32 and TIE(2000)-8 are found to be ~5.8 kHz and ~5.3 kHz, respectively at −60 °C. Decoupling the proton to remove ^7^Li–^1^H dipolar interaction gives the residual linewidths of 0.8 and 1.03 kHz for TIE(2000)-32 and TIE(2000)-8 samples, respectively, which mainly results from ^7^Li–^7^Li homonuclear interaction. Therefore, contributions of ~5.0 kHz for TIE(2000)-32 and ~4.3 kHz for TIE(2000)-8 to the lithium linewidths can be ascribed to ^7^Li–^1^H interaction, *i.e.*, to the interaction between a nearly stationary lithium ion and the protons of the polymer chains. Therefore, approximately 85–80% interaction is for ^7^Li–^1^H and 15–20% for ^7^Li–^7^Li interaction in TIE(2000)-32 and TIE(2000)-8 hybrid electrolyte samples. This confirms that the linewidth of ^7^Li NMR spectra is predominately governed by ^7^Li–^1^H dipolar interactions. These results are comparable to the PEO- and PPO-based SPEs [46,47,48]. At sufficiently high temperatures, each sample exhibits a common high-temperature linewidth limit of about 0.2 kHz. The similarity in the temperature dependence of the linewidth for each sample indicates that a common diffusion mechanism and similar ^7^Li local environments exist for both the samples studied. This suggests that the ions segregate into regions of high density (polymer-rich domain) and low density (silica-rich domain). The change with salt concentration is only associated with the percentages of the two kinds of regions.

The activation energy, *E*_a_, of the samples can be obtained from NMR line narrowing data. The NMR motional narrowing of the ^7^Li linewidth takes place when the rate of fluctuations of the local magnetic fields or electric field gradients, which are generally described by a correlation time, *τ_c_*, is of the order of the rigid lattice linewidth, Δ*_RL_*

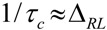
(5)

An estimation of the activation energy for the narrowing process, *E*_a_, may be obtained by the relationship [43]

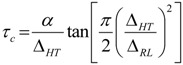
(6)
where Δ*_HT_* and Δ*_RL_* are the FWHHs at a given temperature and in the rigid lattice, respectively, and *α* is a constant of the order of unity. For both the TIE(2000)-32 and TIE(2000)-8 samples, the onset temperature of the rigid lattice linewidth (approximately 5–6 kHz) occurs at about −50 °C. Assuming that *τ_c_* is thermally activated

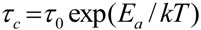
(7)

The *E*_a_ values are calculated from ^7^Li linewidth measurements by fitting equations (6,7) and found to be 0.34 eV and 0.39 eV for the [O]/[Li] ratios of 32 and 8, respectively, which have similarities to the values obtained by other researchers [46,49,50]. Although the measured E_a_ values from the ^7^Li linewidth measurements and the conductivity data (0.32 and 0.42 eV for [O]/[Li] = 32 and 8, respectively) have some variations, more or less the results are similar, which validate the accuracy of the measurement procedure. 

## 3. Experimental Section

### 3.1. Preparation of Hybrid Electrolyte Membranes

Lithium perchlorate (LiClO_4_) and triblock copolymer poly(propylene glycol)-*block*-poly(ethylene glycol)-*block*-poly(propylene glycol) bis(2-aminopropyl ether) (H_2_N-(PPG)_a_(PEG)_b_(PPG)_c_NH_2_, commercially designated by Jeffamine ED2000 (a + c = 3.5 and b = 40.5) and ED600 (a + c = 3.5 and b = 8.5) with *M*_w_ = 2000 and 600 g mol^−1^, respectively) were purchased from Aldrich and dried under vacuum for 3 days prior to use. Since the relative ratios between the ethylene oxide and the propylene oxide units in the polymer play a key role in ion transport, two polymers with a fixed PPG segment but with different lengths of the PEG segment were used. 3-(Triethoxysilyl)propyl isocyanate (ICPTES) (Aldrich) and TEOS (Fluka) were used as received. Synthesis of the organic–inorganic hybrid electrolyte based on H_2_N-(PPG)_a_(PEG)_b_(PPG)_c_NH_2_ triblock copolymer was carried out via a sol-gel route. In a typical synthesis, 2.47 g of ICPTES was first mixed with 0.52 g of TEOS in a molar ratio of 4 *vs.* 1 and then hydrolyzed with 0.5 mL of 2 M HCl at room temperature for 1 h. 5 g of ED2000 or ED600 was dissolved in 15 mL of dried THF and mixed with previous solution and stirred for 24 h. Finally, appropriate amounts of LiClO_4_ were added into the solution to achieve the desired [O]/[Li] ratios and stirred for 24 h at room temperature. The final solution was cast onto Teflon dishes, and the solvent was slowly evaporated at room temperature for 24 h. Afterward, the materials were heated at 80 °C under vacuum for another 24 h to get a transparent and crack-free membrane. The obtained electrolyte membranes were rubbery with good mechanical strength. The membranes were then stored in a glove box under an argon atmosphere for further measurements. The thickness of the membranes was controlled to be in the range of 100–130 µm.

### 3.2. DSC Thermograms

DSC measurements were performed in the temperature range of −60–150 °C using a Perkin-Elmer DSC 6 system at a heating rate of 10 °C min^−1^. The sample weights were maintained in the range of 5–7 mg, and all experiments were carried out under a nitrogen flow. The glass transition temperature (*T*_g_) and melting temperature (*T*_m_) were measured, and the associated enthalpy changes (Δ*H*_f_) were calculated.

### 3.3. FTIR Spectroscopy

FTIR spectra were obtained from a Bio-Rad FTS 155 spectrometer over the range of 4000–400 cm^−1^ at a resolution of 4 cm^−1^ using the KBr wafer technique. Band deconvolution of the resulting spectra was conducted to obtain the best fit for the band envelope.

### 3.4. AC Impedance Measurements

Alternating current (AC) impedance measurements of the hybrid electrolytes were performed using AUTOLAB/PGSTAT 302 frequency response analyzer over a frequency range of 1 Hz to 1 MHz with an amplitude of 10 mV. All the specimens were sandwiched between two polished stainless steel blocking electrodes in argon atmosphere inside a glove box (Innovative technology, PL-HE-2GB with PL-HE-GP1 inert gas purifier) for conductivity tests. These measurements were performed in the temperature range of 15 to 75 °C, and the system was thermally equilibrated at each selected temperature for at least 1 h. The conductivity values (σ) have been calculated from the equation σ = (1/*R*_b_)(*t*/*A*), where *R*_b_ is the bulk resistance, *t* is the thickness and *A* is the area of the sample.

### 3.5. Solid-State NMR Measurements

Solid-state NMR experiments were performed on a Varian Infinityplus-500 NMR spectrometer, equipped with a Chemagnetics 7.5 mm MAS probe and a double tuned static probe. The Larmor frequencies for ^7^Li, ^13^C and ^29^Si nuclei are 194.3, 125.7 and 99.3 MHz, respectively. Magic angle spinning conditions in the range of 3–5 kHz were employed to record ^13^C and ^29^Si NMR spectra. The π/2 pulse lengths for ^7^Li and ^29^Si nuclei were typically 4 and 6 μs, respectively. The ^13^C and ^29^Si chemical shifts were externally referenced to tetramethylsilane (TMS) at 0 ppm. The ^1^H→^13^C cross-polarization magic-angle spinning (CPMAS) NMR spectra were also recorded as a function of CP contact time ranging from 0.2 to 9 ms. ^7^Li NMR spectra were acquired under static conditions with and without proton decoupling during the acquisition. The static ^7^Li line widths were taken to be the full width at half-height of the peaks and measured as a function of temperature from −100 to 100 °C. 

### 3.6. 2D ^1^H–^13^C WISE NMR Experiments

^1^H wide-line spectra were acquired with the use of 2D WISE (two-dimensional WIdeline SEparation spectroscopy) NMR pulse sequence developed by Schmidt-Rohr *et al*. [37]. In the 2D WISE experiments, the proton magnetization evolved under the influence of dipolar coupling during the time *t*_1_, and the ^13^C signal was detected under MAS conditions during the time *t*_2_. The experiment revealed proton wideline spectra from the proton of the polymer chains along the *ω_1_* dimension, resolved by the ^13^C chemical shifts of the polymer chains along the *ω*_2_ dimension. Therefore, a correlation can be made between the chemical structure and segmental mobility of the polymer. Spectral widths of 40 and 100 kHz were used for the *ω*_2_ and *ω*_1_ dimensions, respectively. Typically, 128 *t*_1_ increments were used in the 2D WISE NMR experiments. 

## 4. Conclusions

The effects of lithium salt concentrations, silica domains and molecular weight of the polymers on conductivity, ion structure and dynamics have been investigated in the organic–inorganic hybrid electrolytes based on ED2000/ED600 and complexed with LiClO_4_ via the co-condensation of ICPTES and TEOS. Multinuclear NMR techniques provide a microscopic view for probing the specific interaction between the polymer chain and Li^+^ cation and the behavior of the mobile ionic species, and demonstrate that the segmental mobility of the polymer matrix is affected by the salt concentrations. The combined results revealed that the mobility of charge carriers, low *T*_g_, segmental chain movement, low value of crystallinity and polymer/silica interface layer have contributed in achieving the maximum room temperature ionic conductivity of 5.3 × 10^−5^ Scm^−1^ for TIE(2000)-24 hybrid electrolyte. These important properties make the present hybrid electrolyte membranes of definite interests for the development of solid-state rechargeable lithium batteries. 

## Supplementary Files

Supplementary File 1 PDF-Document (PDF, 185 KB)
