# Supplementary material for: Synthesis, Multinuclear NMR Characterization and Dynamic Property of Organic–Inorganic Hybrid Electrolyte Membrane Based on Alkoxysilane and Poly(oxyalkylene) Diamine
Source: Membranes (Basel). 2012 Jun 13;2(2):253–74. doi: 10.3390/membranes2020253 (PMC4021892; doi:10.3390/membranes2020253)
Supplement: Supplementary File 1 — PDF-Document (PDF, 185 KB) [file membranes-02-00253-s001.pdf]

Membranes **2012**, *2*, 253-274; doi:10.3390/membranes2020253

OPEN ACCESS

membranes

ISSN 2077-0375

www.mdpi.com/journal/membranes

Article

Synthesis, Multinuclear NMR Characterization and Dynamic Property of Organic-Inorganic Hybrid Electrolyte Membrane Based on Alkoxysilane and Poly(oxyalkylene) Diamine

Diganta Saikia, Yu-Chi Pan and Hsien-Ming Kao *

Department of Chemistry, National Central University, Chung-Li 32001, Taiwan;
E-Mails: diganta_s@yahoo.com (D.S.); yuchipan421@gmail.com (Y.-C.P.)

* Author to whom correspondence should be addressed; E-Mail: hmkao@cc.ncu.edu.tw;
Tel.: +886-3-427-5054; Fax: +886-3-422-7664.

Received: 27 April 2012; in revised form: 1 June 2012 / Accepted: 4 June 2012 / Published:

Abstract: Organic-inorganic hybrid electrolyte membranes based on poly(propylene glycol)-*block*-poly(ethylene glycol)-*block*-poly(propylene glycol) bis(2-aminopropyl ether) complexed with $LiClO_4$ via the co-condensation of tetraethoxysilane (TEOS) and 3-(triethoxysilyl)propyl isocyanate have been prepared and characterized. A variety of techniques such as differential scanning calorimetry (DSC), Fourier transform infrared (FTIR) spectroscopy, alternating current (AC) impedance and solid-state nuclear magnetic resonance (NMR) spectroscopy are performed to elucidate the relationship between the structural and dynamic properties of the hybrid electrolyte and the ion mobility. A VTF (Vogel-Tamman-Fulcher)-like temperature dependence of ionic conductivity is observed for all the compositions studied, implying that the diffusion of charge carriers is assisted by the segmental motions of the polymer chains. A maximum ionic conductivity value of 5.3×10^{-5} Scm^{-1} is obtained at 30 ℃. Solid-state NMR results provide a microscopic view of the effects of salt concentrations on the dynamic behavior of the polymer chains.

Keywords: organic-inorganic hybrid electrolyte; ionic conductivity; poly(oxyalkylene) diamine; segmental motion

---

Figure S1. DSC thermograms of TIE(600)-Z hybrid electrolytes with various [O]/[Li] ratios, where Z = (**a**) ∞, (**b**) 32, (**c**) 24, (**d**) 16 and (**e**) 8.

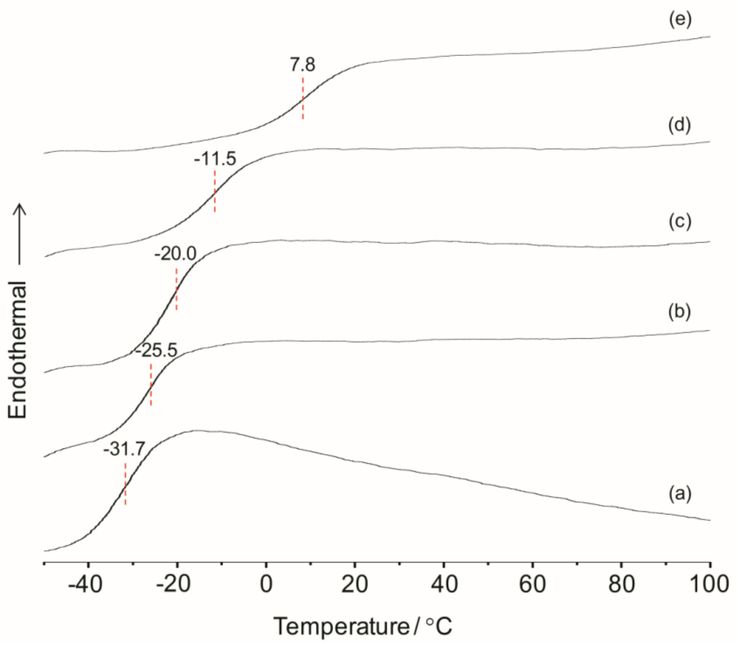

Figure S2. Temperature dependence of ionic conductivity of TIE(600)-Z hybrid electrolytes with Z = (**a**) 32, (**b**) 24, (**c**) 16 and (**d**) 8.

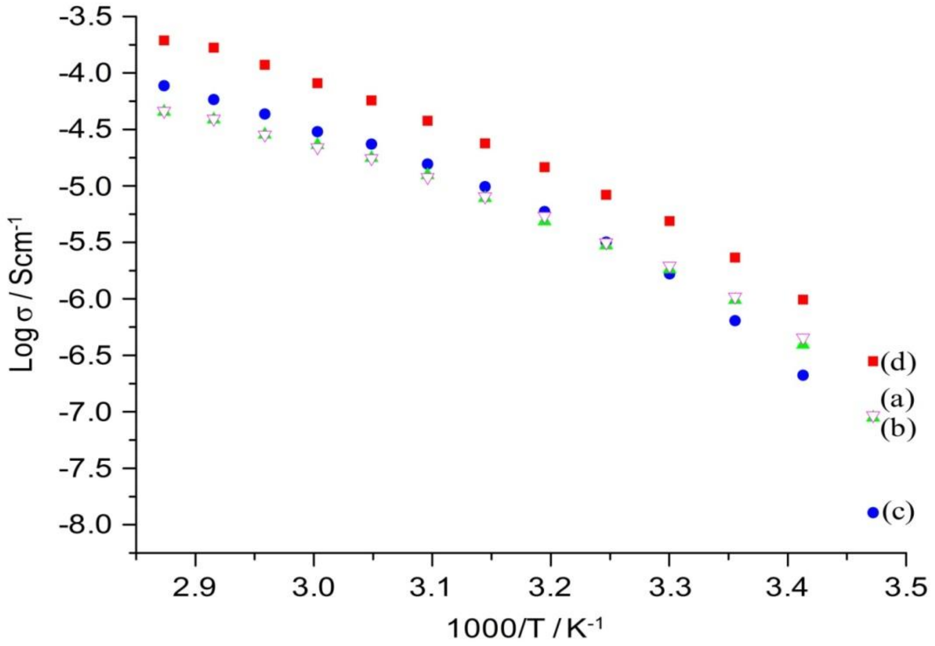

Figure S3. VTF curve fitting results of TIE(2000)-Z hybrid electrolytes with Z = (**a**) 32, (**b**) 24, (**c**) 16 and (**d**) 8.

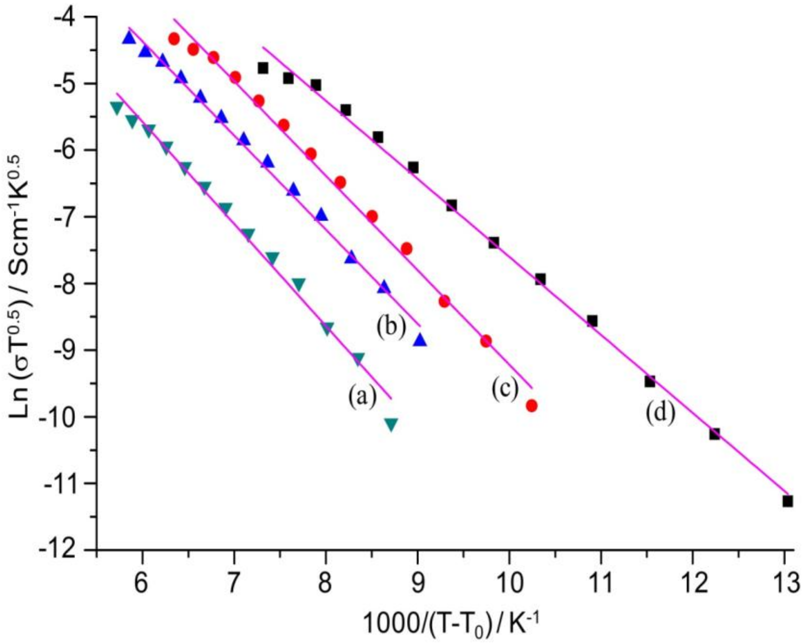

© 2012 by the authors; licensee MDPI, Basel, Switzerland. This article is an open access article distributed under the terms and conditions of the Creative Commons Attribution license (http://creativecommons.org/licenses/by/3.0/).
